# Impact of neoadjuvant chemotherapy and postoperative adjuvant chemotherapy cycles on survival of patients with advanced-stage ovarian cancer

**DOI:** 10.1371/journal.pone.0183754

**Published:** 2017-09-05

**Authors:** Young Shin Chung, Yun-Ji Kim, Inha Lee, Jung-Yun Lee, Eun Ji Nam, Sunghoon Kim, Sang Wun Kim, Young Tae Kim

**Affiliations:** Department of Obstetrics and Gynecology, Institute of Women’s Life Medical Science, Yonsei University College of Medicine, Seoul, Korea; Zhejiang University School of Medicine, CHINA

## Abstract

**Background:**

There is currently no consensus regarding the optimal number of chemotherapy cycles to be administered before and after interval debulking surgery (IDS) in patients with advanced ovarian cancer. This study aimed to evaluate the impact of the number of neoadjuvant chemotherapy (NAC) and postoperative adjuvant chemotherapy (POAC) cycles on the survival of patients with advanced ovarian cancer undergoing NAC/IDS/POAC.

**Methods:**

We retrospectively reviewed data from 203 patients who underwent NAC/IDS/POAC at Yonsei Cancer Hospital between 2006 and 2016. All patients underwent taxane plus carboplatin chemotherapy for NAC and POAC. The patient outcomes were analyzed according to the number of NAC, POAC, and total chemotherapy (NAC+POAC) cycles.

**Results:**

Patients who received fewer than 6 cycles of total chemotherapy (n = 8) had poorer progression-free survival (PFS) and overall survival (OS) than those completing at least 6 cycles (p = 0.005 and p<0.001, respectively). Among patients who completed at least 6 cycles of total chemotherapy (n = 189), Kaplan-Meier analysis revealed no significant difference in either PFS or OS according to the number of NAC cycles (1–3 vs. ≥4; p = 0.136 and p = 0.267, respectively). Among patients who experienced complete remission after 3 cycles of POAC (n = 98), the addition of further POAC cycles did not improve the PFS or OS (3 vs. ≥4; p = 0.641 and p = 0.104, respectively).

**Conclusion:**

IDS after 4 cycles of NAC may be a safe and effective option when completing 6 cycles of total chemotherapy. Furthermore, the addition of more than 3 cycles of POAC does not appear to influence the survival of patients achieving completion remission after 3 cycles of POAC.

## Introduction

Ovarian cancer is the second most common gynecologic cancer in Korea; each year, its incidence and mortality are continuously increasing by 1.5% and 6.2%, respectively [1,2]. The standard treatment for advanced ovarian cancer consists of optimal cytoreductive surgery followed by taxane and platinum-based adjuvant chemotherapy [3]. Neoadjuvant chemotherapy (NAC) and interval debulking surgery (IDS) have been proposed as alternatives for the management of advanced ovarian cancer patients unable to undergo complete debulking during primary surgery [4–6].

Four randomized clinical trials demonstrated that NAC/IDS is not inferior to primary debulking surgery (PDS) for the treatment of advanced ovarian cancer [7–10]. In the European Organization for Research and Treatment of Cancer (EORTC) 55971 trial, patients received 3 cycles of NAC followed by IDS and at least 3 cycles of postoperative adjuvant chemotherapy (POAC) [7]. In the CHORUS trial, patients in the NAC arm underwent 3 cycles of NAC followed by IDS and 3 cycles of POAC [8]. In the SCORPION trial, patients were received 3 or 4 cycles of NAC and then POAC administered after IDS to complete 6 cycles of total chemotherapy [9]. In the JCOG 0602 trial, patients treated 4 cycles of NAC followed by IDS and 4 cycles of POAC [10].

The National Comprehensive Cancer Network (NCCN) guidelines recommend at least a total of 6 cycles of chemotherapy for suspected cases of unresectable advanced-stage ovarian cancer, as well as IDS prior to the fourth cycle of NAC [11]. However, the optimal number of chemotherapy cycles before and after IDS has not been prospectively evaluated, and various clinical regimens are currently utilized based on the patient condition and physician preference. Several studies have demonstrated the safety and feasibility of late IDS, defined as IDS performed after more than 4 cycles of NAC [12–14], whereas others have reported poorer outcomes in patients undergoing late IDS [15,16]. Furthermore, there is currently no evidence that additional chemotherapy of more than 6 cycles of first-line chemotherapy improves outcomes in patients with advanced-stage ovarian cancer [17]. Therefore, the aim of the present study was to evaluate the impact of the number of NAC and POAC cycles on survival in advanced-stage ovarian cancer patients undergoing NAC followed by IDS and POAC.

## Materials and methods

We conducted a retrospective analysis of the medical records of 203 patients with pathologically confirmed epithelial ovarian cancer who received NAC at Yonsei Cancer Hospital between 2006 and 2016. The present study was reviewed and approved by the Institutional Review Board of Severance Hospital at Yonsei University College of Medicine (Registration number: 4-2015-1158) and the need for informed consent was waived. The patient records were anonymized and de-identified prior to analysis.

The inclusion criteria were as follows: (1) pathological diagnosis of International Federation of Gynecology and Obstetrics (FIGO) stage III or IV epithelial ovarian cancer and (2) treatment with at least 1 cycle of NAC followed by IDS. Four patients with FIGO stage I or II ovarian cancer and 2 patients who refused to undergo IDS after NAC were excluded. The remaining 197 patients were included in the final analysis. All surgical procedures were performed by one of five gynecologic oncology surgeons at Yonsei Cancer Hospital, and pathology review was performed by two pathologists at the same institution.

Patients were clinically diagnosed with FIGO stage III or IV by initial imaging workup, comprising abdominal and pelvic computed tomography (CT) and ^18^F-fluorodeoxyglucose-positron emission tomography/CT. The diagnoses were histologically confirmed by examination of the biopsy specimens removed during diagnostic laparoscopy, or by cytological assessment of ascites or pleural effusion. Our institution applied the following principle for the primary treatment strategy: NAC was performed if at least one of the following three criteria was met: 1) pulmonary and/or hepatic parenchymal metastases were observed on imaging studies, 2) the tumors were medically inoperable, and/or 3) optimal cytoreduction was unsuitable due to a high tumor burden (Fagotti score > 8).

Data regarding the patients’ age and body mass index, the FIGO stage, histology, American Society of Anesthesiologists score, preoperative serum cancer antigen-125 level, residual disease after IDS, performance of radical surgery, and number of total chemotherapy, NAC, and POAC cycles were extracted from the patient medical records. All patients received combination chemotherapy with taxane and carboplatin. Conventional surgery included total hysterectomy, bilateral salpingo-oophorectomy, infracolic omentectomy, pelvic/para-aortic lymph node dissection, and appendectomy. Radical surgery was defined as operations involving radical oophorectomy, bowel resection, diaphragm or other peritoneal surface stripping, splenectomy, partial hepatectomy, partial gastrectomy, or partial cystectomy and/or ureteroneocystostomy, cholecystectomy, and/or distal pancreatectomy [18–20]. CT images were obtained prior to IDS and after 3 cycles of POAC to evaluate the patient responses to treatment. Objective responses were determined based on the Response Evaluation Criteria in Solid Tumors (RECIST) [21]. Survival outcomes were analyzed according to the number of total chemotherapy (NAC+POAC; <6 vs. 6 vs. ≥7), NAC (1–3 vs. ≥4), and POAC (3 vs. ≥4) cycles.

Overall survival (OS) was defined as the time from the date of first chemotherapy to the date of death. Progression-free survival (PFS) was defined as the time from the date of first chemotherapy to the date of first recurrence. The treatment-free interval (TFI) was defined as the interval from the end of platinum-based chemotherapy to the first recurrence.

### Statistical analysis

SPSS version 18 for Windows (SPSS, Inc., Chicago, IL, USA) was used for all statistical analyses. Survival curves were constructed using the Kaplan-Meier method, with the log-rank test applied to detect differences between the groups. Multivariate analyses for OS and PFS were performed using Cox regression models. We investigated the linear trends in the number of NAC/POAC cycles using the chi-square test. For all analyses, the level of statistical significance was set at p < 0.05.

## Results

A total of 197 patients with pathologically confirmed FIGO stage III or IV epithelial ovarian cancer underwent at least 1 cycle of NAC followed by IDS at our institution during the study period. The baseline characteristics of all patients are detailed in Table 1. The median patient age was 57 years (range: 27–80 years). We confirmed the diagnosis histologically by diagnostic laparoscopy in 119 (60.4%), by cytology of ascites in 56 (28.4%) or pleural effusion in 22 (11.2%) before starting chemotherapy. The percentage of patients with FIGO stage IV ovarian cancer at the time of diagnosis was 73.6%. To achieve maximum cytoreduction, 84 (42.64%) patients underwent at least one radical surgery, including bowel surgery at the time of IDS in 30 patients (15.23%). The percentage of patients with no gross residual disease following cytoreduction was 36.6%. The median number of total chemotherapy cycles (NAC+POAC) was 8 (range: 3–12), while the median number of NAC cycles was 3 (range: 1–9), and the median number of POAC cycles was 5 (range: 0–9). All patients received first-line taxane plus platinum combination chemotherapy as NAC and POAC. After NAC, most patients (n = 160, 81.2%) exhibited a partial response, and 98 (49.7%) patients exhibited a complete response after 3 cycles of POAC.

**Table 1:** Baseline patient characteristics (n = 197).

Variables	
Median age, years (range)	57 (27–80)
ASA score, n (%)	
1	51 (26.3)
2	92 (47.4)
3	50 (25.8)
4	1 (0.5)
Not available	3 (1.5)
Median BMI at diagnosis, kg/m2 (range)	22.83 (16.38–35.84)
Median CA 125 level at diagnosis, U/mL (range)	1825.7 (44.3–30000)
FIGO stage, n (%)	
IIIB	7 (3.6)
IIIC	45 (22.8)
IVA	89 (45.2)
IVB	56 (28.4)
Histologic type, n (%)	
Serous	180 (91.4)
Mucinous	4 (2.0)
Endometrioid	3 (1.5)
Clear cell	7 (3.6)
Others	3 (1.5)
Radical surgery, n (%)	
None	113 (57.36)
Bowel surgery	30 (15.23)
VATS	29 (14.72)
Splenectomy	25 (12.69)
Liver resection	17 (8.63)
SCF resection	4 (2.03)
Breast/axillar LN dissection	6 (3.05)
Ureter resection	4 (2.03)
Others	14 (7.11)
Methods of biopsy, n (%)	
Diagnostic laparoscopy	119 (60.4)
Ascites	56 (28.4)
Pleural effusion	22 (11.2)
Residual disease after IDS, n (%)	
NGR	72 (36.6)
≤0.5 cm	63 (32.0)
≤1 cm	27 (13.7)
≤2	5 (2.5)
>2	8 (4.1)
Unknown	22 (11.2)
Cycles of NAC, median (range)	3 (1–9)
Number of NAC cycles, n (%)	
<4	152 (77.2)
≥4	45 (22.8)
Cycles of POAC, median (range)	5 (0–9)
Number of POAC cycles, n (%)	
<4	83 (42.1)
≥4	114 (57.9)
Cycles of total chemotherapy (NAC+POAC), median (range)	8 (3–12)
Number of total chemotherapy (NAC+POAC), n (%)	
<6	8 (4.1)
≥6	189 (95.9)

ASA, American Society of Anesthesiologists; BMI, body mass index; CA 125, cancer antigen 125; FIGO, Federation of Gynecology and Obstetrics; VATS, video-assisted thoracoscopic surgery; SCF, supraclavicular fossa; LN, lymph node; NGR, no gross residual disease; NAC, neoadjuvant chemotherapy; POAC, postoperative adjuvant chemotherapy

The median follow-up was 26.7 months (range: 3.0–101.8 months), during which there were 131 recurrences and 71 deaths. Eight patients underwent <6 cycles of total chemotherapy (NAC+POAC); the reasons for discontinuation in these patients are shown in Table 2.

**Table 2:** Reasons for not completing 6 cycles of total chemotherapy (n = 8).

	n (%)
Progression of disease	3 (37.5)
Poor general condition	2 (25.0)
Death	2 (25.0)
Follow up loss	1 (12.5)

**Fig 1:**
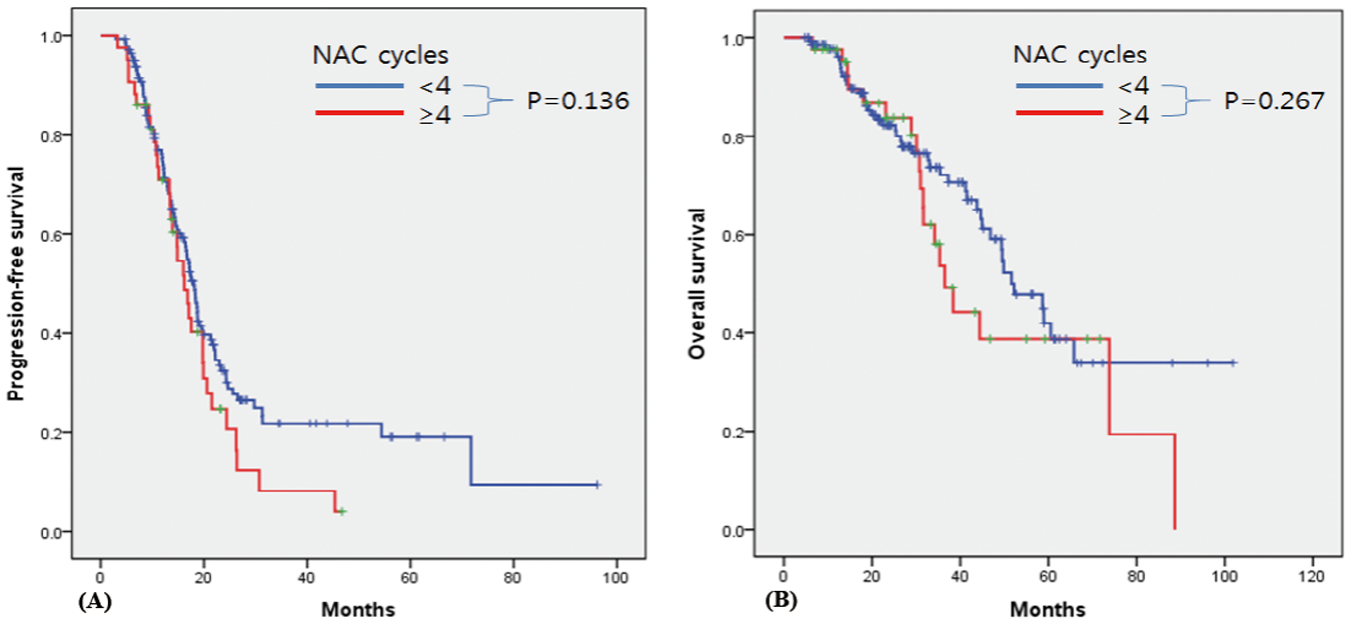
Kaplan-Meier survival curves for patients undergoing at least 6 cycles of total chemotherapy. (A) Progression-free survival and (B) overall survival according to the number of NAC cycles (<4 vs. ≥4). NAC: neoadjuvant chemotherapy.

**Fig 2:**
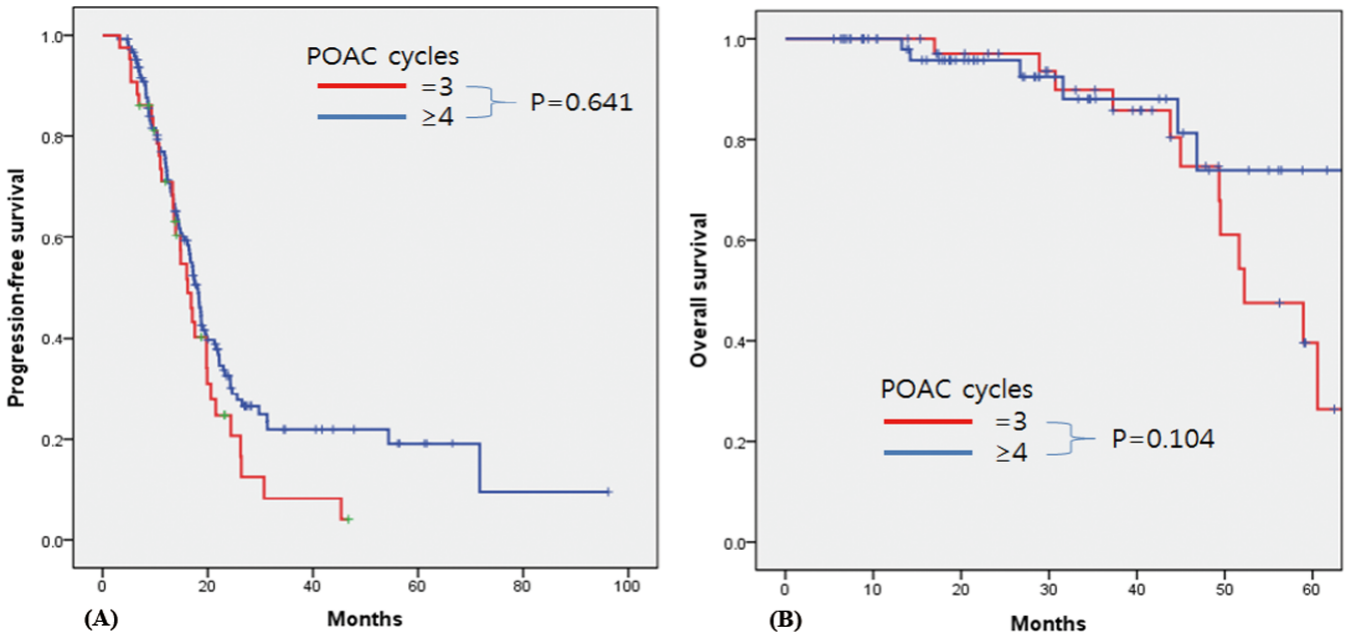
Kaplan-Meier survival curves for patients who attained complete remission after 3 cycles of POAC and underwent at least 6 cycles of total chemotherapy. (A) Progression-free survival and (B) overall survival according to the number of additional POAC cycles (3 vs. ≥4). POAC: postoperative adjuvant chemotherapy.

Compared to patients receiving 6 and ≥7 cycles of total chemotherapy, patients who received <6 cycles (n = 8) showed worse PFS and OS (p = 0.005 and p<0.001, respectively) (S1 Fig). Based on this finding, we next analyzed the correlation between the number of NAC/POAC cycles and clinical outcomes in patients who completed at least 6 cycles of total chemotherapy. Among patients who underwent at least 6 cycles of total chemotherapy, Kaplan-Meier curve and log-rank analyses revealed no significant difference in either PFS or OS between patients undergoing IDS after 1–3 vs. ≥4 cycles of NAC (p = 0.136 and p = 0.267, respectively) (Fig 1). After 3 cycles of POAC, 98 patients experienced complete remission, as determined by CT. Among these patients, the use of more than 3 cycles of POAC did not significantly improve the PFS or OS (3 vs. ≥4 cycles, p = 0.641 and p = 0.104, respectively; Fig 2). Overall, the results were consistent when we performed subgroup analysis for patients with high-grade serous carcinoma (HGSC) (S2–S4 Figs). When we performed sensitivity analysis in patients who completed 6 cycles and 9 cycles of total chemotherapy, there was no significant difference in either PFS or OS between patients undergoing IDS after 1–3 vs. ≥4 cycles of NAC in patients (S5 and S6 Figs). In addition, when we confined to patients who underwent 3 cycles of NAC, the additional of more than 3 cycles of POAC did not significantly improve the outcome (S7 Fig).

**Fig 3:**
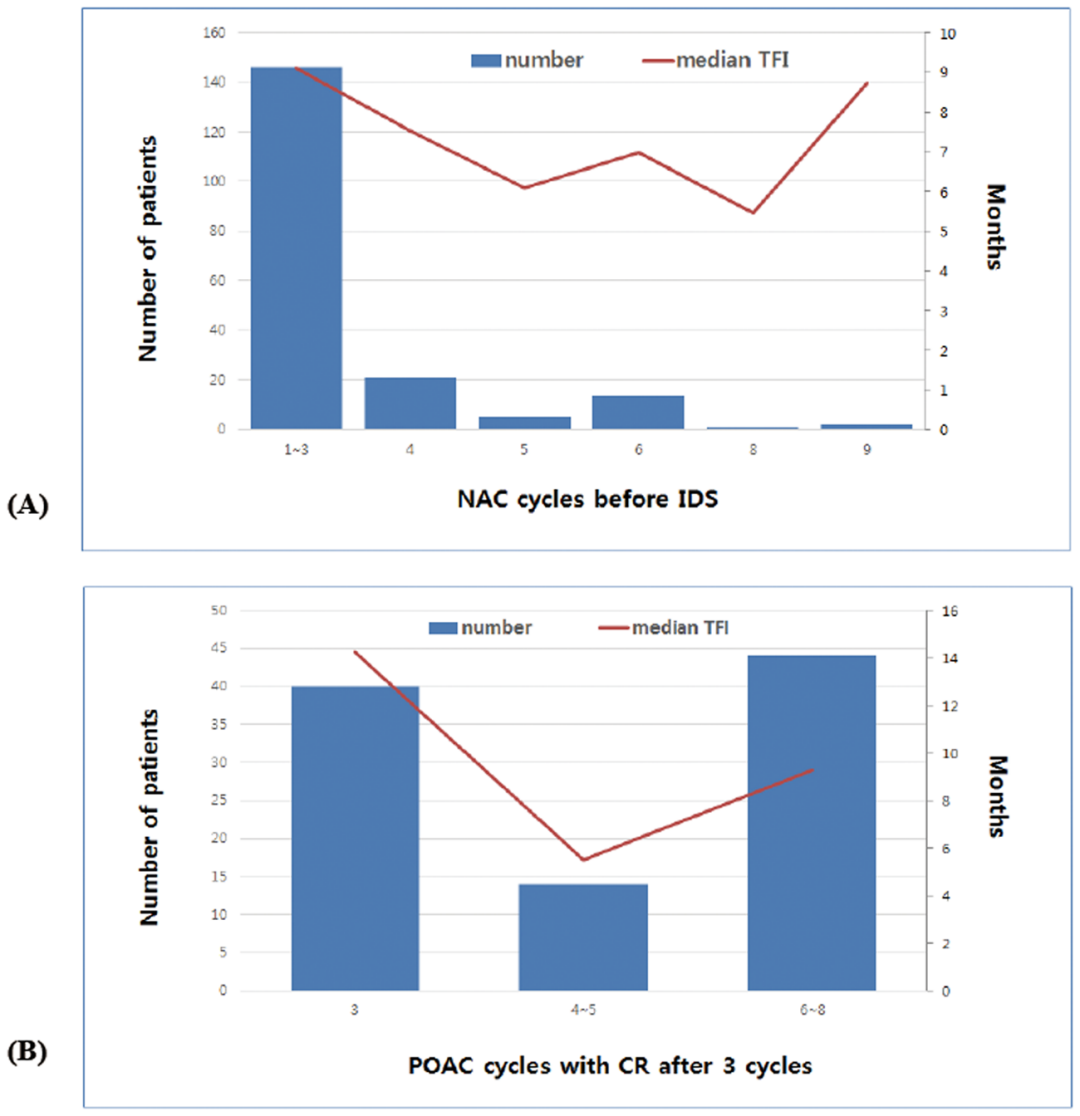
Linear trends analyses for the median TFI as the number of NAC/POAC cycles increases. The tendency of the median TFI according to (A) the timing of interval debulking surgery and (B) the number of POAC cycles in patients with complete remission after 3 cycles. TFI: treatment-free interval; NAC: neoadjuvant chemotherapy; POAC: postoperative adjuvant chemotherapy.

Next, we evaluated the relationship between the median TFI and the number of NAC cycles before IDS to determine the optimal timing of IDS (Fig 3A). We also evaluated the correlation between the median TFI and the number of POAC cycles in patients with complete remission after 3 cycles of POAC to determine the most appropriate number of POAC cycles (Fig 3B). No significant correlation was observed between the risk of recurrence and the number of NAC cycles before IDS or the number of POAC cycles in patients with complete remission after 3 cycles of POAC (p for trend = 0.386 and 0.215, respectively).

Multivariate analyses revealed a significant effect of the number of total chemotherapy cycles (NAC+POAC) on both PFS (hazard ratio 0.36; 95% confidence interval, 0.15–0.84) and OS (hazard ratio, 0.09; 95% confidence interval, 0.03–0.26) in patients who had undergone at least 6 cycles (S1 Table). However, among these patients, neither the number of NAC cycles nor POAC cycles influenced the PFS or OS (S2 Table).

## Discussion

In the present study, we aimed to evaluate the association between the number of NAC/POAC/total chemotherapy cycles and clinical outcomes in patients with advanced ovarian cancer. Our findings suggest that completion of at least 6 cycles of total chemotherapy is an independent prognostic factor in patients undergoing NAC/IDS/POAC. However, the prescription of more than 3 cycles of NAC or POAC did not significantly improve the patient survival further.

The ideal time for cytoreductive surgery in patients undergoing NAC/IDS remains controversial. The NCCN guidelines recommend IDS prior to the fourth cycle of NAC. The evidence for 3 cycles of NAC is based on the findings of two randomized controlled trials, which demonstrated that such treatment was equivalent in efficacy to PDS [7,8]. The protocols of these two trials specified that IDS should be performed after 3 cycles of NAC. Other recent studies have reported conflicting results for performing late IDS after >4 cycles of NAC in patients with advanced ovarian cancer [12–16]. In one previous study, IDS after >6 cycles of NAC was found to be both safe and effective, with outcomes equivalent to those reported for the standard treatment [13]. Akladios et al. reported that the number of NAC cycles did not affect the survival of advanced-stage ovarian cancer patients [12], while Colombo et al. reported that patients who received >4 cycles of NAC before IDS experienced poor prognosis, despite optimal debulking surgery. Similarly, Xu et al. reported that patients who underwent >5 cycles of NAC experienced shorter PFS than patients who underwent <5 cycles [16].

The appropriate number of POAC cycles after IDS also remains controversial. Although 3–6 cycles of POAC are typically administered, various protocols are utilized based on the patient’s condition and physician preference. The EORTC trial recommended at least 3 cycles of POAC after IDS [7], whereas the CHORUS and SUNNY trials specified that precisely 3 cycles of POAC should be administered after IDS [8]. There is limited evidence suggesting that the addition of >3 cycles of POAC after IDS improves outcomes in patients with advanced-stage ovarian cancer. Xu et al. reported that patients who underwent <5 cycles of adjuvant chemotherapy after IDS experienced shorter PFS and OS than patients who underwent >5 cycles of adjuvant chemotherapy after IDS [16]. Prolonged cycles of POAC may be useful in patients with residual tumors or an incomplete response after 3 cycles of POAC. Therefore, in the present study, we analyzed the outcomes in a subgroup of patients who achieved complete radiological remission after 3 cycles of POAC. However, our analysis revealed no benefit of additional POAC cycles.

Our study has several implications. Our findings suggest that patients with advanced ovarian cancer should be treated with at least 6 cycles of total chemotherapy (NAC+POAC). Indeed, we observed that patients treated with <6 cycles of total chemotherapy experienced poorer PFS and OS than those treated with ≥6 cycles of total chemotherapy. These findings suggest the importance of adhering to the current guidelines, which recommend the use of at least 6 cycles of total chemotherapy for the treatment of advanced-stage ovarian cancer [22].

We further observed that the number of NAC cycles did not influence the survival of the patients, suggesting that the neoadjuvant approach with late IDS is safe and effective for patients who are unable to undergo complete debulking surgery. Indeed, we observed similar survival outcomes for this approach and IDS after 3 cycles of NAC. Furthermore, the addition of >3 POAC cycles after IDS did not improve the PFS and OS in patients who had achieved complete radiological remission after 3 cycles of POAC. It is important to note that additional cycles not only increase the cost of treatment, but also increase the risk of toxicity and platinum resistance due to prolonged chemotherapy. In a previous study involving patients who had achieved complete remission after PDS plus 6 cycles of chemotherapy, the addition of 3 cycles of consolidation chemotherapy did not improve survival outcomes, although increased toxicity was observed [23]. Similarly, addition of >3 cycles of POAC did not improve the survival outcomes in patients achieving complete remission after NAC/IDS plus 3 cycles of POAC in the present study. Therefore, additional maintenance chemotherapy should be regarded as an optional, rather than a necessary, treatment strategy, as determined based on the individual patient’s condition [23–26].

The present study also possesses several limitations. First, as this was a retrospective study, selection bias was inevitable and may have influenced our results. Especially, the timing of IDS was decided at the treating physician’s discretion. After each cycle of NAC, each gynecologic oncologist evaluated the patient’s status and recommended further NAC in individuals who were not suitable for complete debulking and still exhibited a poor response. Second, with the increased use of NAC in recent years, our cohort is limited by the short follow-up period. Further studies to obtain long-term follow-up data on the PFS and OS rates are needed.

In conclusion, our findings indicate that completion of at least 6 cycles of total chemotherapy is an independent prognostic factor in patients undergoing NAC/IDS/POAC. Although such treatment is currently recommended, we observed that the addition of more than 3 NAC or POAC cycles did not affect the patient survival. Therefore, IDS after more than 4 cycles of NAC may be a safe and effective option for patients unable to undergo optimal cytoreduction. Furthermore, our findings indicate that additional POAC cycles may not be necessary when complete remission is achieved after 3 cycles of POAC.

## Supporting information

S1 Fig Kaplan-Meier survival curves. (A) Progression-free survival and (B) overall survival according to the number of total (NAC+POAC) chemotherapy cycles (<6 vs. 6 vs. ≥7). NAC: neoadjuvant chemotherapy; POAC: postoperative adjuvant chemotherapy.
(TIF)

S2 Fig Kaplan-Meier survival curves for patients with HGSC. (A) Progression-free survival and (B) overall survival according to the number of total (NAC+POAC) chemotherapy cycles (<6 vs. 6 vs. ≥7). NAC: neoadjuvant chemotherapy; POAC: postoperative adjuvant chemotherapy; HGSC: high-grade serous carcinoma.
(TIF)

S3 Fig Kaplan-Meier survival curves for patients undergoing at least 6 cycles of total chemotherapy with HGSC. (A) Progression-free survival and (B) overall survival according to the number of NAC cycles (<4 vs. ≥4). NAC: neoadjuvant chemotherapy; HGSC: high-grade serous carcinoma.
(TIF)

S4 Fig Kaplan-Meier survival curves for patients who attained complete remission after 3 cycles of POAC and underwent at least 6 cycles of total chemotherapy with HGSC. (A) Progression-free survival and (B) overall survival according to the number of additional POAC cycles (3 vs. ≥4). POAC: postoperative adjuvant chemotherapy; HGSC: high-grade serous carcinoma.
(TIF)

S5 Fig Sensitivity analysis in subgroup of patients who completed 6 cycles of total chemotherapy. (A) Progression-free survival and (B) overall survival according to the number of NAC cycles (<4 vs. ≥4). NAC: neoadjuvant chemotherapy.
(TIF)

S6 Fig Sensitivity analysis in subgroup of patients who completed 9 cycles of total chemotherapy. (A) Progression-free survival and (B) overall survival according to the number of NAC cycles (<4 vs. ≥4). NAC: neoadjuvant chemotherapy.
(TIF)

S7 Fig Kaplan-Meier survival curves for patients underwent 3 cycles of NAC and at least 6 cycles of total chemotherapy when complete remission was achieved after 3 cycles of POAC. (A) Progression-free survival and (B) overall survival according to the number of additional POAC cycles (3 vs. ≥4). NAC: neoadjuvant chemotherapy; POAC: postoperative adjuvant chemotherapy.
(TIF)

S1 Table Univariate and multivariate analyses for progression-free survival and overall survival. (DOCX)

S2 Table Univariate and multivariate analyses for progression-free survival and overall survival in patients undergoing at least 6 cycles of total chemotherapy. (DOCX)
