# Supplementary figures and images for: Impact of neoadjuvant chemotherapy and postoperative adjuvant chemotherapy cycles on survival of patients with advanced-stage ovarian cancer
Source: PLoS One. 2017 Sep 5;12(9):e0183754. doi: 10.1371/journal.pone.0183754 (PMC5584794; doi:10.1371/journal.pone.0183754)

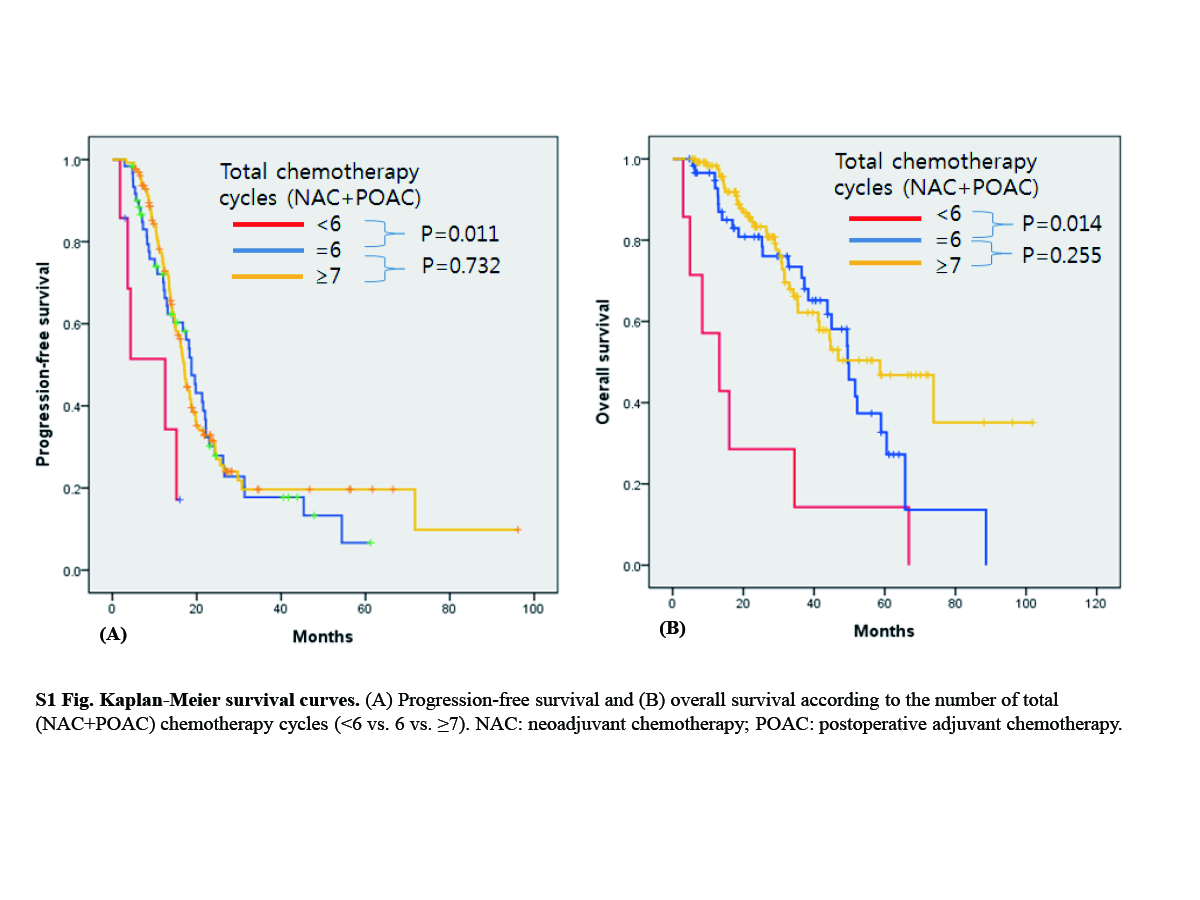

Supplement: S1 Fig — (A) Progression-free survival and (B) overall survival according to the number of total (NAC+POAC) chemotherapy cycles (<6 vs. 6 vs. ≥7). NAC: neoadjuvant chemotherapy; POAC: postoperative adjuvant chemotherapy. (TIF) [file pone.0183754.s001.tif]

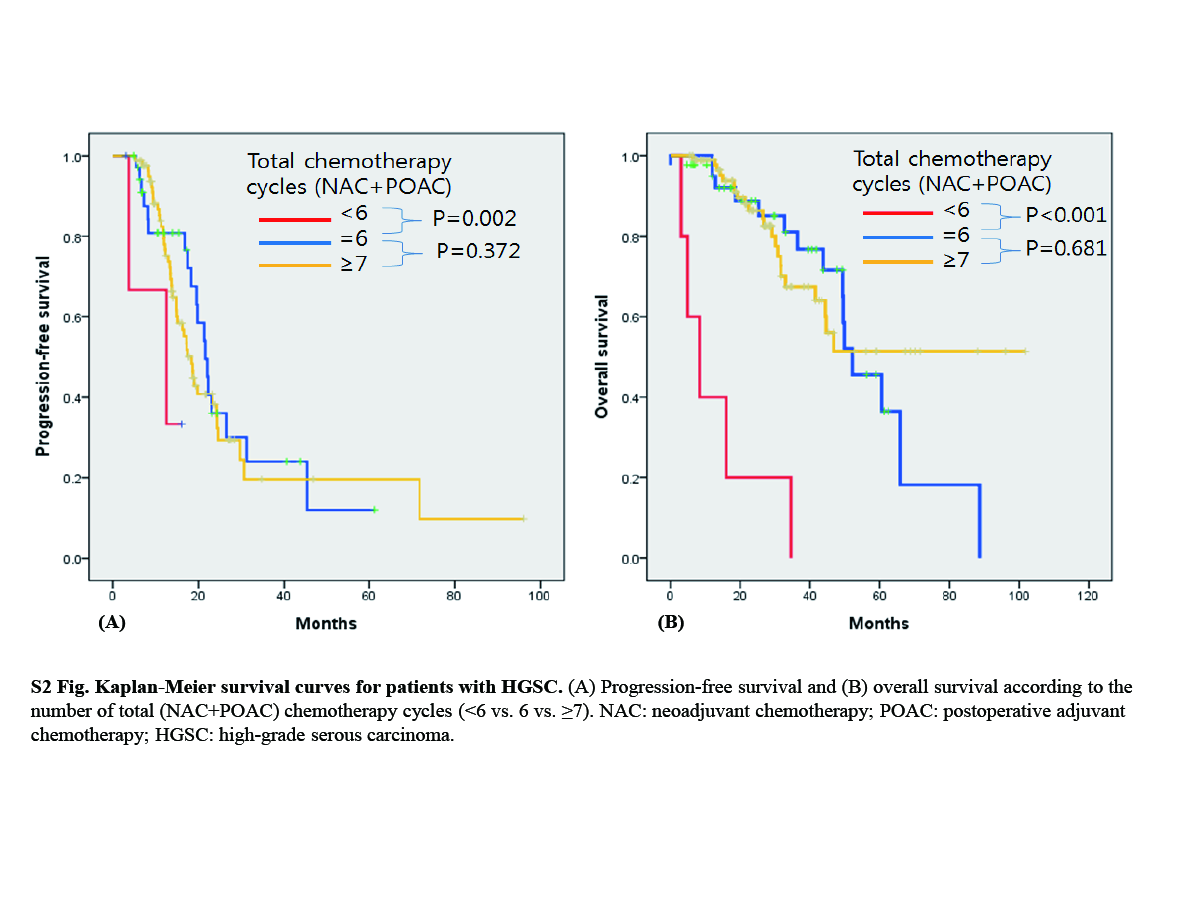

Supplement: S2 Fig — (A) Progression-free survival and (B) overall survival according to the number of total (NAC+POAC) chemotherapy cycles (<6 vs. 6 vs. ≥7). NAC: neoadjuvant chemotherapy; POAC: postoperative adjuvant chemotherapy; HGSC: high-grade serous carcinoma. (TIF) [file pone.0183754.s002.tif]

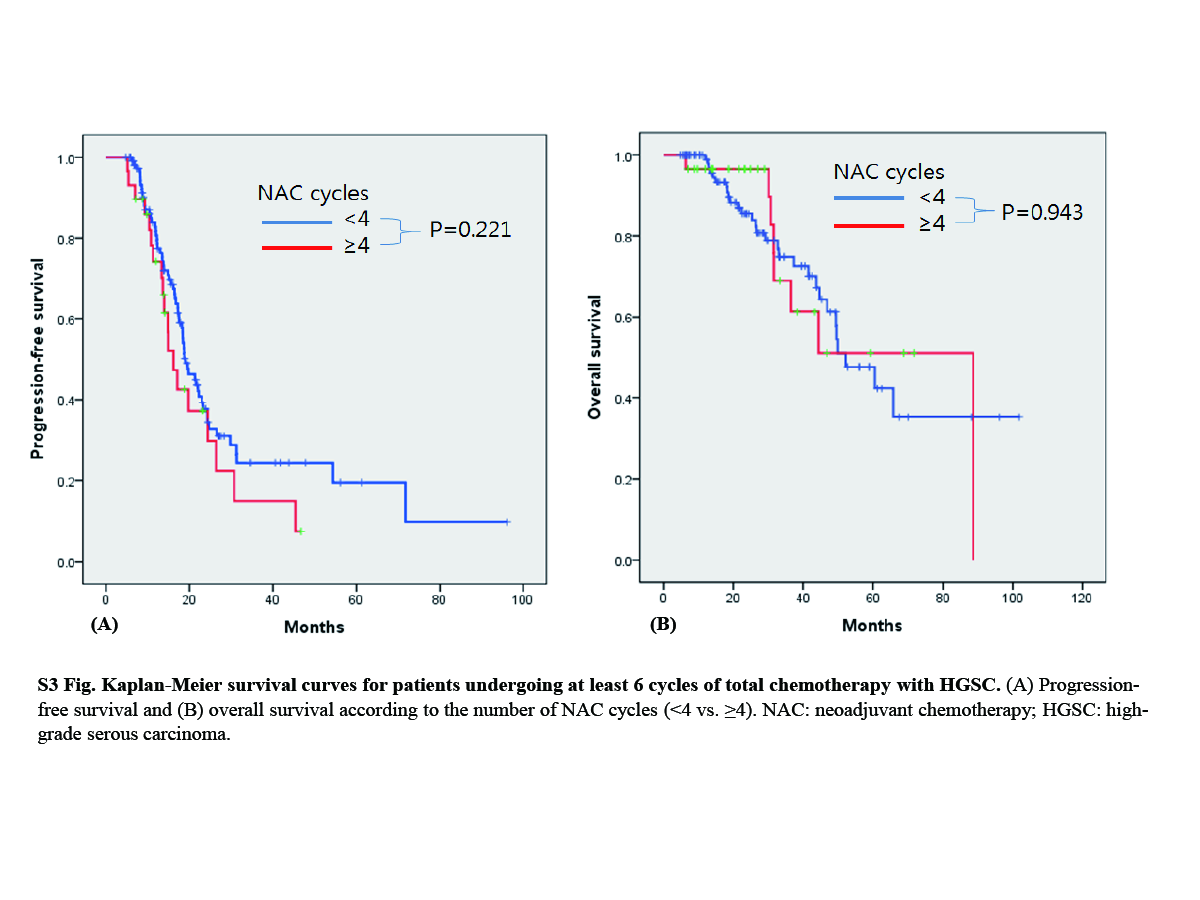

Supplement: S3 Fig — (A) Progression-free survival and (B) overall survival according to the number of NAC cycles (<4 vs. ≥4). NAC: neoadjuvant chemotherapy; HGSC: high-grade serous carcinoma. (TIF) [file pone.0183754.s003.tif]

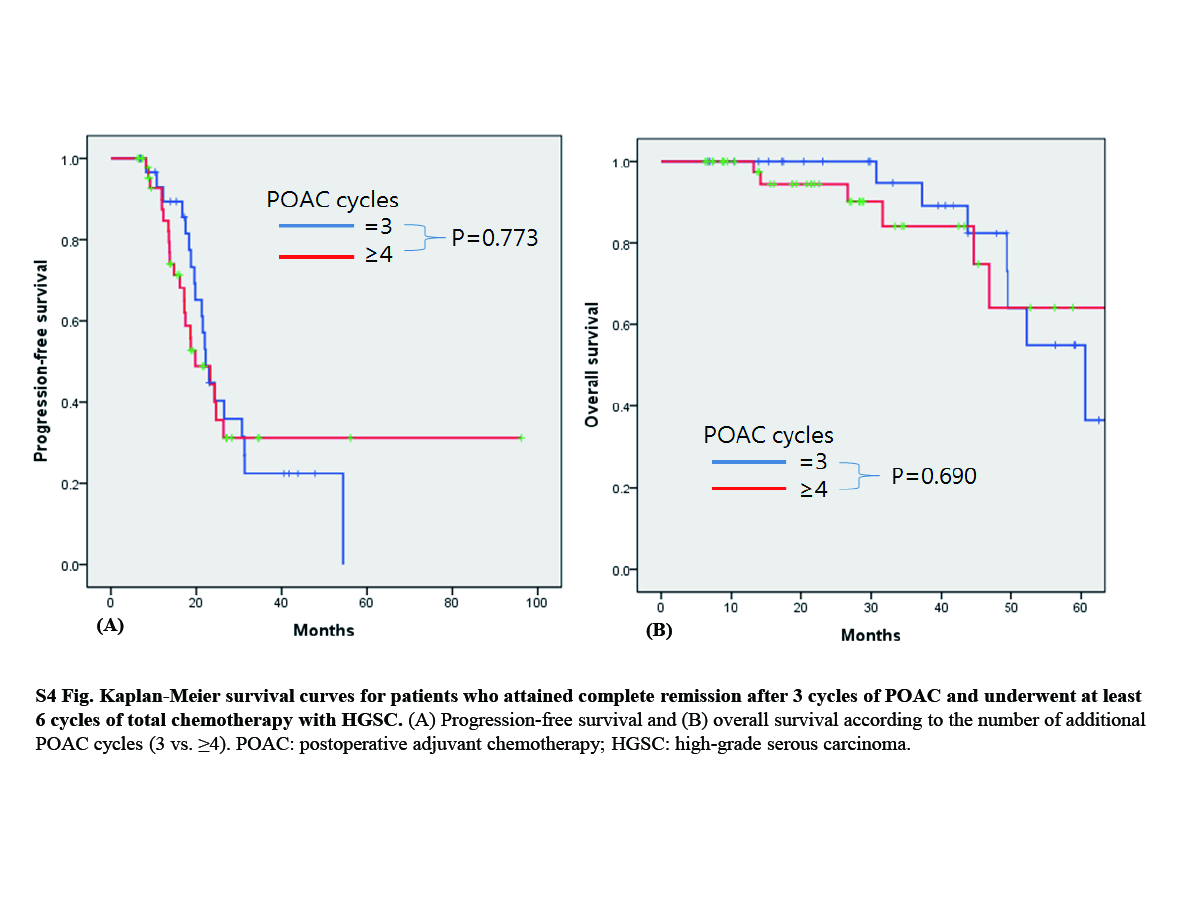

Supplement: S4 Fig — (A) Progression-free survival and (B) overall survival according to the number of additional POAC cycles (3 vs. ≥4). POAC: postoperative adjuvant chemotherapy; HGSC: high-grade serous carcinoma. (TIF) [file pone.0183754.s004.tif]

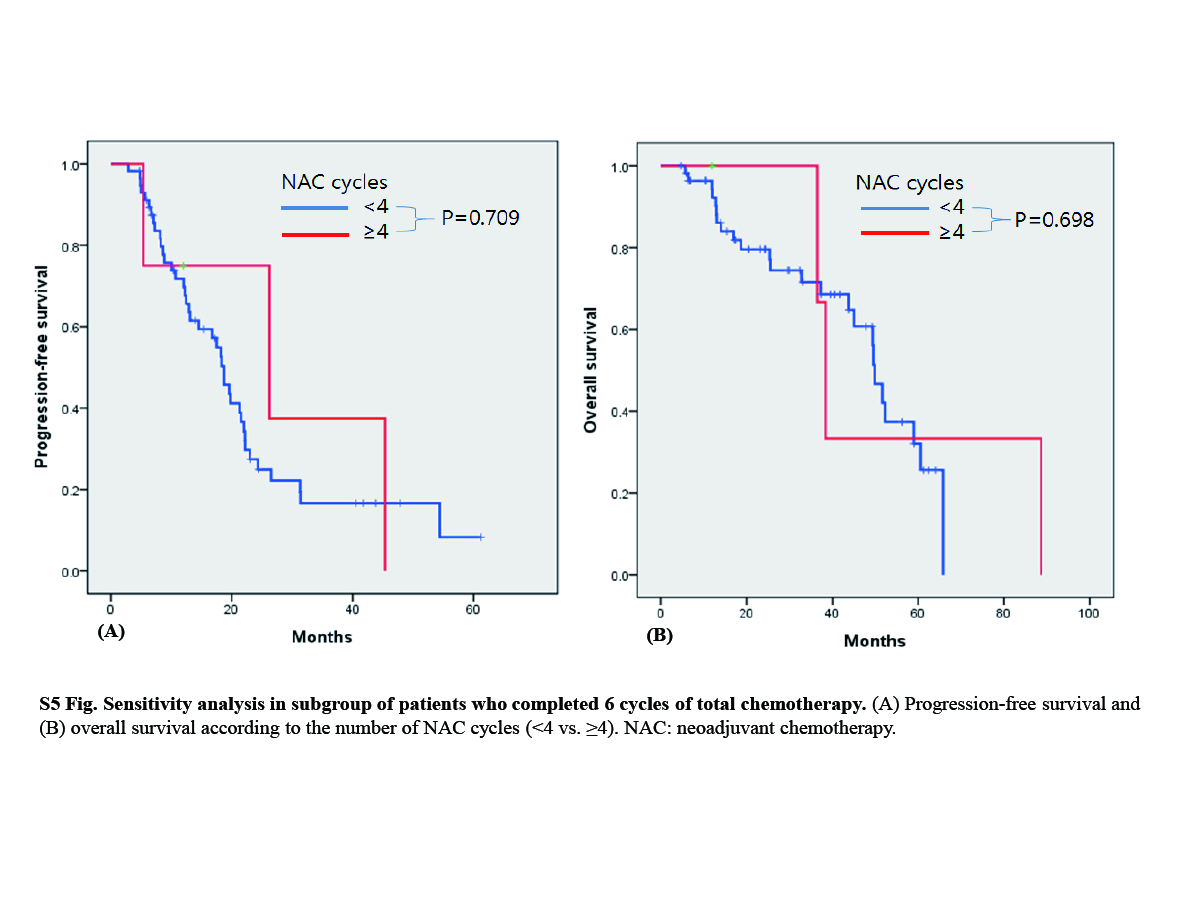

Supplement: S5 Fig — (A) Progression-free survival and (B) overall survival according to the number of NAC cycles (<4 vs. ≥4). NAC: neoadjuvant chemotherapy. (TIF) [file pone.0183754.s005.tif]

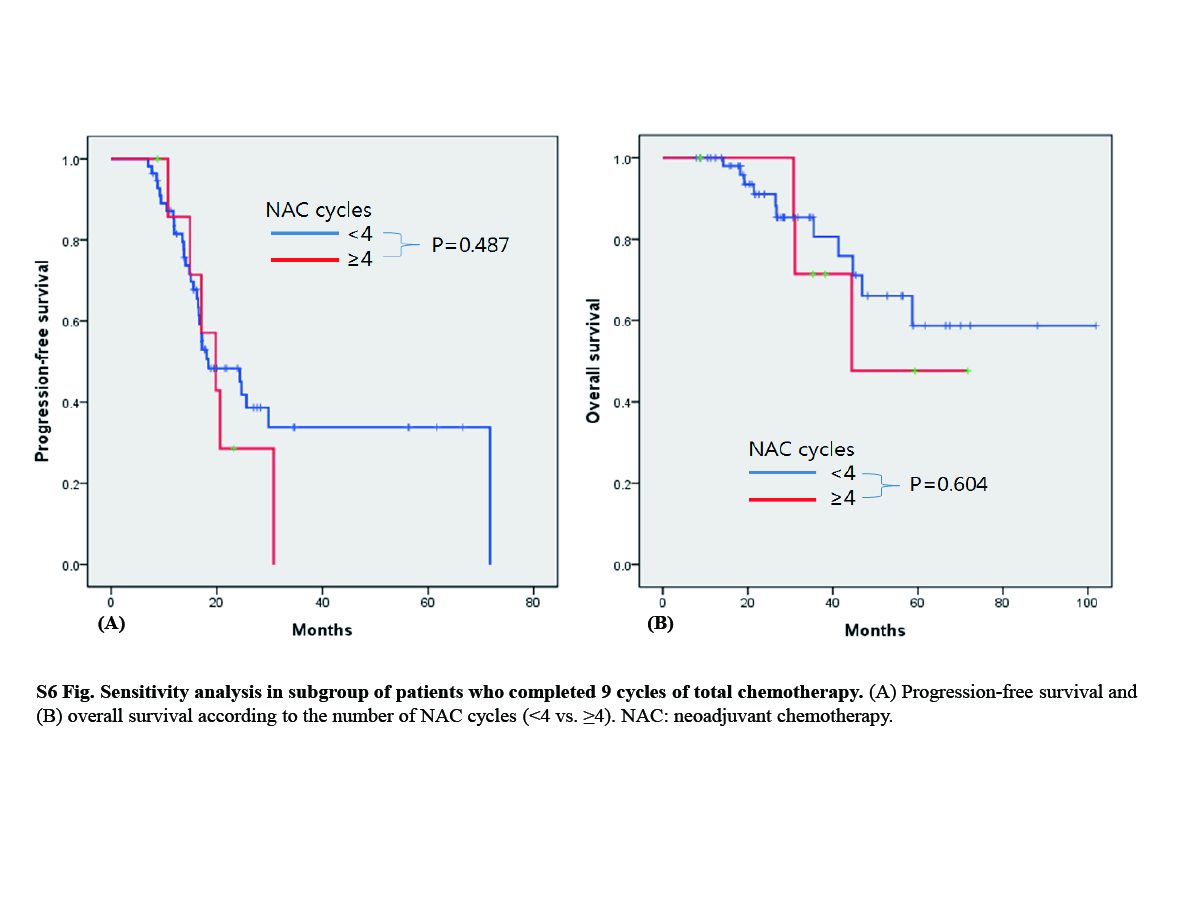

Supplement: S6 Fig — (A) Progression-free survival and (B) overall survival according to the number of NAC cycles (<4 vs. ≥4). NAC: neoadjuvant chemotherapy. (TIF) [file pone.0183754.s006.tif]

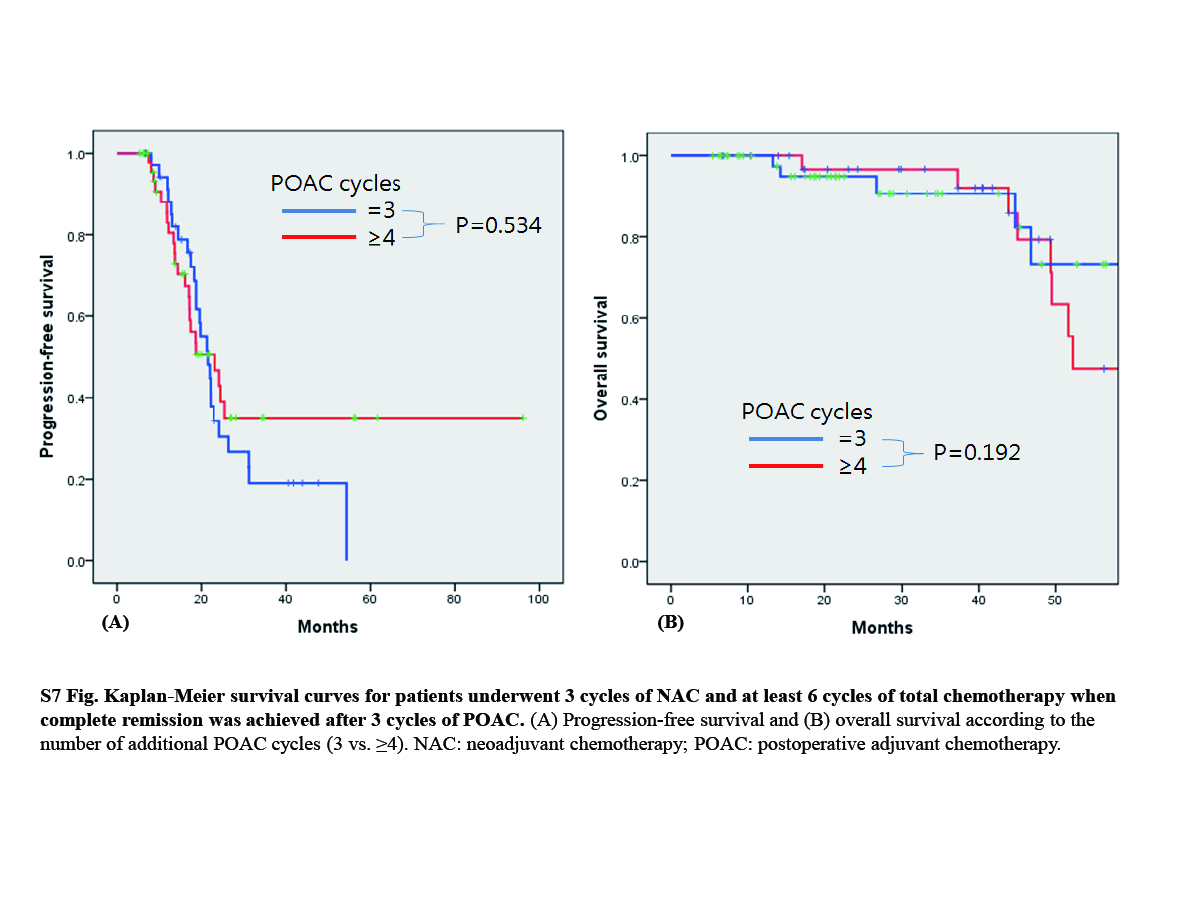

Supplement: S7 Fig — (A) Progression-free survival and (B) overall survival according to the number of additional POAC cycles (3 vs. ≥4). NAC: neoadjuvant chemotherapy; POAC: postoperative adjuvant chemotherapy. (TIF) [file pone.0183754.s007.tif]
