# Supplementary material for: Impact of neoadjuvant chemotherapy and postoperative adjuvant chemotherapy cycles on survival of patients with advanced-stage ovarian cancer
Source: PLoS One. 2017 Sep 5;12(9):e0183754. doi: 10.1371/journal.pone.0183754 (PMC5584794; doi:10.1371/journal.pone.0183754)
Supplement: S1 Table — (DOCX) [file pone.0183754.s008.docx]

S1 Table. Univariate and multivariate analyses for progression-free survival and overall survival

| Variables |  | PFS | |  | OS | |
| --- | --- | --- | --- | --- | --- | --- |
|  |  | Univariate analysis | Multivariate analysis |  | Univariate analysis | Multivariate analysis |
|  |  | HR (95% CI) | HR (95% CI) |  | HR (95% CI) | HR (95% CI) |
| Age (years) |  |  |  |  |  |  |
| <60 |  | 1 (Reference) |  |  | 1 (Reference) | 1 (Reference) |
| ≥60 |  | 1.05 (0.68-1.62) |  |  | 1.72 (0.99-2.99) | 1.82 (1.07-3.12) |
| ASA |  |  |  |  |  |  |
| 1-2 |  | 1 (Reference) |  |  | 1 (Reference) |  |
| 3-4 |  | 1.24 (0.75-2.03) |  |  | 1.58 (0.74-3.36) |  |
| FIGO stage |  |  |  |  |  |  |
| III |  | 1 (Reference) | 1 (Reference) |  | 1 (Reference) |  |
| IV |  | 1.76 (1.06-2.91) | 1.82 (1.16-2.96) |  | 0.91 (0.48-1.70) |  |
| Histology |  |  |  |  |  |  |
| Non-HGSC |  | 1 (Reference) | 1 (Reference) |  | 1 (Reference) | 1 (Reference) |
| HGSC |  | 0.42 (0.23-0.79) | 0.43 (0.23-0.78) |  | 0.27 (0.12-0.59) | 0.26 (0.12-0.55) |
| Residual disease |  |  |  |  |  |  |
| NGR |  | 1 (Reference) | 1 (Reference) |  | 1 (Reference) | 1 (Reference) |
| Any residual |  | 1.73 (1.14-2.64) | 1.74(1.15-2.65) |  | 2.92 (1.45-5.89) | 3.05 (1.49-6.23) |
| Surgery extent |  |  |  |  |  |  |
| Standard |  | 1 (Reference) |  |  | 1 (Reference) | 1 (Reference) |
| Radical |  | 1.12 (0.71-1.76) |  |  | 1.79 (0.93-3.42) | 1.79 (0.95-3.35) |
| Bowel surgery |  |  |  |  |  |  |
| No |  | 1 (Reference) |  |  | 1 (Reference) | 1 (Reference) |
| Yes |  | 0.94 (0.48-1.83) |  |  | 0.23 (0.08-0.67) | 0.23 (0.08-0.69) |
| Number of total chemotherapy cycles |  |  |  |  |  |  |
| <6 |  | 1 (Reference) | 1 (Reference) |  | 1 (Reference) | 1 (Reference) |
| ≥6 |  | 0.41 (0.16-1.08) | 0.36 (0.15-0.84) |  | 0.10 (0.04-0.28) | 0.09 (0.03-0.26) |
| PFS, progression-free survival; OS, overall survival; HR, hazard ratio; CI, confidence interval; ASA, American Society of Anesthesiologists; FIGO, Federation of Gynecology and Obstetrics; HGSC, high-grade serous carcinoma; NGR, no gross residual disease | | | | | | |
