# Supplementary material for: Impact of neoadjuvant chemotherapy and postoperative adjuvant chemotherapy cycles on survival of patients with advanced-stage ovarian cancer
Source: PLoS One. 2017 Sep 5;12(9):e0183754. doi: 10.1371/journal.pone.0183754 (PMC5584794; doi:10.1371/journal.pone.0183754)
Supplement: S2 Table — (DOCX) [file pone.0183754.s009.docx]

S2 Table. Univariate and multivariate analyses for progression-free survival and overall survival in patients undergoing at least 6 cycles of total chemotherapy

| Variables |  | PFS | |  | OS | |
| --- | --- | --- | --- | --- | --- | --- |
|  |  | Univariate analysis | Multivariate analysis |  | Univariate analysis | Multivariate analysis |
|  |  | HR (95% CI) | HR (95% CI) |  | HR (95% CI) | HR (95% CI) |
| Age (years) |  |  |  |  |  |  |
| <60 |  | 1 (Reference) |  |  | 1 (Reference) | 1 (Reference) |
| ≥60 |  | 1.18 (0.71-1.95) |  |  | 2.20 (1.17-4.13) | 2.20 (1.18-4.12) |
| FIGO stage |  |  |  |  |  |  |
| III |  | 1 (Reference) | 1 (Reference) |  | 1 (Reference) |  |
| IV |  | 1.72 (1.00-3.01) | 1.56 (0.93-2.63) |  | 0.78 (0.37-1.63) |  |
| Histology |  |  |  |  |  |  |
| Non-HGSC |  | 1 (Reference) | 1 (Reference) |  | 1 (Reference) | 1 (Reference) |
| HGSC |  | 0.35 (0.18-0.69) | 0.35 (0.18-0.67) |  | 0.24 (0.10-0.57) | 0.21 (0.09-0.47) |
| Residual disease |  |  |  |  |  |  |
| NGR |  | 1 (Reference) | 1 (Reference) |  | 1 (Reference) | 1 (Reference) |
| Any residual |  | 1.93 (1.21-3.06) | 1.82(1.16-2.84) |  | 3.33 (1.39-7.95) | 3.28 (1.38-7.81) |
| Surgery extent |  |  |  |  |  |  |
| Standard |  | 1 (Reference) |  |  | 1 (Reference) | 1 (Reference) |
| Radical |  | 0.97 (0.59-1.60) |  |  | 2.06 (0.97-4.35) | 1.88 (0.95-3.71) |
| Bowel surgery |  |  |  |  |  |  |
| No |  | 1 (Reference) |  |  | 1 (Reference) | 1 (Reference) |
| Yes |  | 1.15 (0.56-2.36) |  |  | 0.27 (0.08-0.91) | 0.28 (0.09-0.85) |
| Number of NAC cycles |  |  |  |  |  |  |
| <4 |  | 1 (Reference) |  |  | 1 (Reference) |  |
| ≥4 |  | 1.48 (0.91-2.42) |  |  | 1.15 (0.57-2.30) |  |
| Number of POAC cycles |  |  |  |  |  |  |
| 3 |  | 1 (Reference) |  |  | 1 (Reference) |  |
| ≥4 |  | 0.70 (0.46-1.07) |  |  | 0.84 (0.46-1.55) |  |
| PFS, progression-free survival; OS, overall survival; HR, hazard ratio; CI, confidence interval; ASA, American Society of Anesthesiologists; FIGO, Federation of Gynecology and Obstetrics; HGSC, high-grade serous carcinoma; NGR, no gross residual disease; NAC, neoadjuvant chemotherapy; POAC, postoperative adjuvant chemotherapy | | | | | | |
